# Combining Multiple Approaches and Models to Dissect the Genetic Architecture of Resistance to Infections in Fish

**DOI:** 10.3389/fgene.2020.00677

**Published:** 2020-07-10

**Authors:** Clémence Fraslin, Edwige Quillet, Tatiana Rochat, Nicolas Dechamp, Jean-Francois Bernardet, Bertrand Collet, Delphine Lallias, Pierre Boudinot

**Affiliations:** ^1^INRAE, AgroParisTech, GABI, Université Paris-Saclay, Jouy-en-Josas, France; ^2^INRAE, UVSQ, VIM, Université Paris-Saclay, Jouy-en-Josas, France

**Keywords:** fish, disease resistance, QTL mapping, transcriptomics, host–pathogen interactions, immunity, functional assays, fish isogenic lines

## Abstract

Infectious diseases represent a major threat for the sustainable development of fish farming. Efficient vaccines are not available against all diseases, and growing antibiotics resistance limits the use of antimicrobial drugs in aquaculture. It is therefore important to understand the basis of fish natural resistance to infections to help genetic selection and to develop new approaches against infectious diseases. However, the identification of the main mechanisms determining the resistance or susceptibility of a host to a pathogenic microbe is challenging, integrating the complexity of the variation of host genetics, the variability of pathogens, and their capacity of fast evolution and adaptation. Multiple approaches have been used for this purpose: (i) genetic approaches, QTL (quantitative trait loci) mapping or GWAS (genome-wide association study) analysis, to dissect the genetic architecture of disease resistance, and (ii) transcriptomics and functional assays to link the genetic constitution of a fish to the molecular mechanisms involved in its interactions with pathogens. To date, many studies in a wide range of fish species have investigated the genetic determinism of resistance to many diseases using QTL mapping or GWAS analyses. A few of these studies pointed mainly toward adaptive mechanisms of resistance/susceptibility to infections; others pointed toward innate or intrinsic mechanisms. However, in the majority of studies, underlying mechanisms remain unknown. By comparing gene expression profiles between resistant and susceptible genetic backgrounds, transcriptomics studies have contributed to build a framework of gene pathways determining fish responsiveness to a number of pathogens. Adding functional assays to expression and genetic approaches has led to a better understanding of resistance mechanisms in some cases. The development of knock-out approaches will complement these analyses and help to validate putative candidate genes critical for resistance to infections. In this review, we highlight fish isogenic lines as a unique biological material to unravel the complexity of host response to different pathogens. In the future, combining multiple approaches will lead to a better understanding of the dynamics of interaction between the pathogen and the host immune response, and contribute to the identification of potential targets of selection for improved resistance.

## Introduction

The outcome of infections is determined by multiple factors including the direct tissue damages caused by the pathogenic microbe (or parasite), the capacity of the immune system to prevent or limit its multiplication, and the adverse effects of the inflammation induced by their confrontation. Infectious diseases exert powerful selection pressures on host populations, affecting survival, growth, and fertility of the host. Host–pathogen interactions are therefore shaped by a dynamic co-evolution of the virulence factors of the pathogen and the defense mechanisms of the host. Within the same host species, this co-evolution contributes to marked differences in susceptibility between host populations from disease-free areas or from areas where the pathogen is present (Hedrick et al., 2003). Emergent diseases caused by pathogens that have recently crossed a host species barrier are often devastating because their virulence has not been attenuated by these evolutionary processes. At the population level, it is important to note that commensalism is not always the ideal final equilibrium, and that fast transmission or high durability of the pathogen can promote an evolution of host–parasite relationships toward a state of severe disease (Ewald, 1983).

Multiple anti-infectious host defense mechanisms control the pathogenesis. They can act at every step of host–pathogen interactions, from the entry into host organism/cell to the production and dissemination of the pathogen. All mechanisms of immunity can potentially affect the resistance or susceptibility to a given pathogen. Furthermore, many other (i.e., non-immune) mechanisms like virus receptors or tissue tropism may affect the efficiency of the pathogen biology or the beneficial/harmful result of the host response.

The identification of the main mechanisms determining the resistance or susceptibility of a host to a pathogenic microbe is therefore extremely challenging, integrating the complexity of the variation of host genetics and the variability of pathogens and their capacity of fast evolution and adaptation. Genetic approaches such as QTL (quantitative trait loci) mapping make it possible to disentangle these complex interactions by providing information on the genetic determinism of host resistance and, ultimately, on the underlying genetic variations (and thus mechanisms) that make a host resistant or susceptible (Houston et al., 2020). Functional assays (comparative transcriptome analysis, *in vitro* culture models) also provide insights into mechanisms of interaction between the pathogen and its host and can help in identifying genes that play a key role in host response to infection. Combining such positional and functional approaches is very promising, as exemplified by the identification of genes involved in intrinsic restriction of retroviruses: the gene Fv1 (Friend-virus susceptibility gene-1) responsible for the susceptibility of mice to Murine Leukemia Virus was identified by Stoye and colleagues using a positional cloning strategy (Best et al., 1996), while the gene *trim5* responsible for the resistance of rhesus cells to HIV-1 was cloned in parallel using a cDNA expression library by direct selection of virus-resistant transfected cells (Stremlau et al., 2004). In this work, we did not address the interactions between fish resistance or susceptibility, and the variation of virulence within pathogen species. It is certainly an important—and understudied—issue, but the discussion of these mechanisms is beyond the scope of the present work.

Infectious diseases remain a major threat for the development and the economic and environmental efficiency of fish farming. Bacterial diseases can be treated by antibiotics, but such treatments lead to the development of resistant microbes, which reduce treatment efficiency and represent a significant issue for animal and human health. Vaccines can efficiently protect fish against infectious diseases, and indeed allowed a drastic reduction of antibiotic treatments in Nordic salmon aquaculture. However, vaccines are not available against all diseases. Moreover, they are generally efficient when administered by injection, which is not possible for small individuals. There are no vaccines against many viral diseases and no vaccine protecting against fish parasites (Collins et al., 2019; Ma et al., 2019). Recent discoveries on probiotics raise hope for beneficial adjustment of gut microbiota, but no such treatment has been fully validated to date (Conti et al., 2014). Hence, genetic selection of fish with improved resistance to the main infectious diseases in a given environment remains a highly sought-after objective in aquaculture (Houston et al., 2020).

Fish lifestyle in aquaculture conditions has an important impact on the interactions between farmed fish and their pathogens. The concentrations of animals in cages or small water bodies allow major outbreaks (Lafferty et al., 2015). Also, this enhances the transmission efficiency, hence allowing pathogens to evolve higher virulence and pathogenicity. Importantly, as for other farmed species, “domestication” and selection for positive traits such as fast growth, food efficiency etc. at the industrial scale of modern aquaculture has led to a genetic homogenization of fish stocks and may have been detrimental for resistance to (at least some) pathogens.

In a context of globalization leading to severe problems due to invasive species (including pathogens), selection of resistance to diseases that are important locally may not be sufficient on the long term. The production of robust fish constituting interesting compromises between specific resistances and a general capacity to deal with multiple aggressors might be the ultimate aim.

Tolerance, i.e., the ability to limit pathogenesis of a given pathogen burden, is another important parameter of fish/pathogen interactions and survival, which shows genetic variability within animal populations (Råberg et al., 2007). Only a few reports have been published on such mechanisms in fish. For example, increased tolerance to viral infection by chikungunya virus has been shown in zebrafish in which the *inos* gene had been knocked down (Levraud et al., 2014). Since both mechanisms and genetic variability of tolerance remains poorly known in fish, this review does not cover this subject.

**FIGURE 1:**
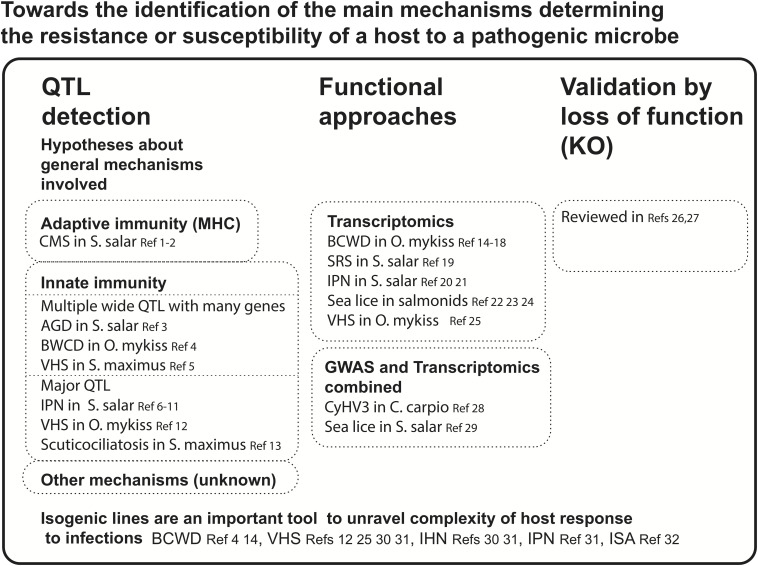
Toward the identification of the main mechanisms determining the resistance or susceptibility of a host to a pathogenic microbe. CMS, cardiomyopathy syndrome; AGD, amoebic gill disease; BCWD, bacterial cold water disease; VHS, viral hemorrhagic septicemia; IPN, infectious pancreatic necrosis; INH, infectious hematopoietic necrosis; ISA, infectious salmon anemia; SRS, salmonid rickettsial septicemia; CyHV-3, cyprinid herpesvirus; QTL, quantitative trait loci; GWAS, genome-wide association study. References: ^1^Boison et al., 2019a; ^2^Hillestad and Moghadam, 2019; ^3^Boison et al., 2019b; ^4^Fraslin et al., 2018; ^5^Rodríguez-Ramilo et al., 2014; ^6^Houston et al., 2008; ^7^Houston et al., 2010; ^8^Houston et al., 2012; ^9^Gheyas et al., 2010; ^10^Moen et al., 2009; ^11^Moen et al., 2015; ^12^Verrier et al., 2013a; ^13^Saura et al., 2019; ^14^Langevin et al., 2012; ^15^Marancik et al., 2015; ^16^Paneru et al., 2016; ^17^Zwollo et al., 2017; ^18^Moore et al., 2018; ^19^Dettleff et al., 2015; ^20^Reyes-López et al., 2015; ^21^Robledo et al., 2016; ^22^Sutherland et al., 2014; ^23^Braden et al., 2015; ^24^Holm et al., 2015; ^25^Verrier et al., 2018; ^26^Dehler et al., 2019; ^27^Gratacap et al., 2019; ^28^Palaiokostas et al., 2018b; ^29^Robledo et al., 2019; ^30^Verrier et al., 2012; ^31^Verrier et al., 2013b; ^32^Biacchesi et al., 2007^32^.

In this review, we focus on the different approaches developed to better understand and take benefit from resistance mechanisms against pathogens in fish (Figure 1). We first discuss examples of identification of QTL of resistance to pathogenic microbes. We then focus on transcriptomics studies and functional assays that describe host–pathogen interactions. The cross information from these two approaches allows a better understanding of the dynamics of interaction between the pathogen and the host immune response and contributes to the identification of potential targets of selection for improved resistance. Finally, we emphasize the importance of combining several approaches (QTL mapping and resequencing or gene expression) and provide a few perspectives on new approaches (such as eQTL, reQTL, and other combination of QTL with expression studies) that should help to identify the genes responsible for resistance to pathogens in fish.

## Describing the Genetic Architecture of Resistance Traits in Fish to Identify Underlying Genetic Mechanisms

### Mapping of QTL Associated With Disease Resistance in Fish

One approach to better understand the host–pathogen interaction in fish is to describe the genetic architecture of traits related to host response to diseases. The detection and mapping of QTL associated with resistance traits after infections provides information along the genome on the position and effect of genetic variants associated with a trait of interest. QTL mapping relies on preferential association between known molecular genetic markers (microsatellites or SNPs) and an unknown causative mutation that affect the trait. The two main types of analysis used to detect QTL are (1) linkage analysis (LA) in full or half-sib families with low-density markers spread throughout the genome and (2) genome-wide association studies (GWAS) performed in large populations using thousands of SNPs. In fish, numerous QTL studies aiming at describing the genetic architecture of disease-related traits have been published in the past 20 years with an intensification since 2013: we found more than 60 studies reporting QTL associated with different resistance traits in 16 fish species infected with 30 pathogens (see Tables 1, 2 and Supplementary Table 1).

**TABLE 1:** Summary of the relevant QTL studies pointing toward the implication of adaptive mechanisms in disease resistance.

Fish species	Disease	Challenge type	Experimental design (genotyped fish in the analysis)	Genetic molecular markers	Resistance traits	Number of detected QTL	Size of detected QTL	% of variance explained by QTL	Publication
Atlantic salmon (*S. salar*)	CMS	Injection (ip) and natural outbreak	571 and 4312 and 901 (FS and HF in three populations)	55,735 SNPs	BS and lesion score	2 QTL	0.2–31 Mb	8–31% of Vg	Boison et al., 2019a
Atlantic salmon (*S. salar*)	CMS	Injection (ip)	1144 fish (60 FS)	48K SNPs	Lesion score and viral load	2 QTL	320 kb (Ssa12) and 19 Mb (Ssa27)	Up to 57% of Vg (Ssa27)	Hillestad and Moghadam, 2019
Atlantic salmon (*S. salar*)	Sea lice	Immersion	94 fish (3 FS)	SSR markers located within 3′ of MHC I (in two chromosomes)	Parasite count	2 QTL	28 cM	13% of Vp	Gharbi et al., 2009
Atlantic salmon (*S. salar*)	ISA	Injection (ip)	1031 fish (25 FS)	17 polymorphic repeats located within 3′ of MHC I and MHC II	BS	5 MHC I and II alleles			Grimholt et al., 2003
Atlantic salmon (*S. salar*)	Furunculosis	Cohabitation	1182 fish (33 FS)	17 polymorphic repeats located within 3′ of MHC I and MHC II	BS	7 MHC I and II alleles			Grimholt et al., 2003
Rainbow trout (*O. mykiss*)	IPN	Immersion	199 fish (BC and F1)	226 markers (microsatellites AFPLs, MHC I and II genes)	BS	9 QTL		8–15% of Vp	Ozaki et al., 2007
Turbot (*S. maximus*)	Scuticociliatosis	Injection (ic)	758 fish (4 FS)	211 microsatellites	BS and TTD	5–10 QTL	7–30 cM	7–22% of Vp	Rodríguez-Ramilo et al., 2013
Red sea bream (*P. major*)	RSI disease	Injection	120 and 400 fish (2 F1)	458 microsatellites	BS	1 QTL		12–31% of Vp	Sawayama et al., 2017

*QTL, quantitative trait loci; CMS, cardiomyopathy syndrome; ISA, infectious salmon anemia; IPN, infectious pancreatic necrosis; RSI, red seabream iridovirus; ip, intraperitoneal; ic, intracelomical; BS, binary survival that takes into account the outcomes of the challenge as dead or alive; TTD, time to death, measured as the time (in days or hours) between the infection and the day of death of a fish; Vp, phenotypic variance of the resistance trait; Vg, genetic or genomic variance of the resistance trait; FS; full-sib; HS, half-Sib; BC, back-cross; SNP, single-nucleotide polymorphism; MHC, major histocompatibility complex; cM, centi-Morgan. Size of detected QTL corresponds to either confidence intervals when calculated or cumulated windows with significant SNPs.*

**TABLE 2:** Summary of the relevant QTL studies pointing toward the implication of innate or intrinsic mechanisms in disease resistance.

Fish species	Disease	Challenge type	Experimental design (genotyped fish in the analysis)	Genetic molecular markers	Resistance traits	Number of detected QTL	Size of detected QTL	% variance explained by QTL	Publication
Atlantic salmon (*S. salar*)	AGD	Immersion	1333 fish (136 FS)	53,109 SNPs	Lesion score	3 QTL + smaller QTL	6 kb–1.2 Mb	4.6–5.3% of Vg	Boison et al., 2019b
Rainbow trout (*O. mykiss*)	BCWD	Immersion, injection (im)	DH fish 195 for immersion, 115 for injection	2130 SNPs	BS, TTD, COX endurance	15 QTL	20–80 cM	7–18% of Vp	Fraslin et al., 2018
Atlantic salmon (*S. salar*)	IPN	Natural outbreak	584 fish (10 FS)	Less than 100 microsatellites	BS	4 QTL	10–69 cM	8.9–24.6% of Vp	Houston et al., 2008
Atlantic salmon (*S. salar*)	IPN	Immersion	1321 fish (10 FS)	7 microsatellites	BS and TTD	3 QTL	10 cM	50.9% of Vp for Ssa26	Houston et al., 2010
Atlantic salmon (*S. salar*)	IPN	Immersion	28 fish (2 FS) and 9,000 fish (400 FS)	6712 SNPs	BS	1 QTL	2 cM		Houston et al., 2012
Atlantic salmon (*S. salar*)	IPN	Immersion	634 fry (20 FS) over 2 mph	8 microsatellites	BS	1 major QTL on Ssa26	7 cM	0–43.9% of Vp	Gheyas et al., 2010
Atlantic salmon (*S. salar*)	IPN	Cohabitation	1896 fish (10 FS) at 10 mph	136 microsatellites	BS	3 QTL with 1 major QTL	4 cM	29% of Vp 83% of Vg	Moen et al., 2009
Atlantic salmon (*S. salar*)	IPN	Immersion	4140 fish (207 FS)	3 microsatellites	BS	1 major QTL on Ssa26			Moen et al., 2015
Turbot (*S. maximus*)	Scuticociliatosis	Cohabitation	1394 fish (36 FS)	18,125 SNPs	Resistance Endurance Resilience	4 QTL: 1 major and 3 minors	Up to 9.3 Mb	Up to 33% Vg	Saura et al., 2019
Turbot (*S. maximus*)	VHS	Injection (ic)	280 fish (3 FS)	93 microsatellites	BS and TTD	7 QTL	4–30 cM	3–14% Vp	Rodríguez-Ramilo et al., 2014
Rainbow trout (*O. mykiss*)	VHS	Immersion	1325 DH fish + 450 DH fish (VREFT)	131–142 microsatellites	VREFT and COX	7 QTL (1 major)	1 cM	Up to 65% of Vp	Verrier et al., 2013a

*AGD, amoebic gill disease; BCWD, bacterial cold water disease; IPN, infectious pancreatic necrosis; VHS, viral hemorrhagic septicemia; BS, binary survival that takes into account the outcomes of the challenge as dead or alive; TTD, time to death, measured as the time (in days or hours) between the infection and the day of death of a fish; COX, survival analysis with a Cox model (Cox, 1972); VREFT, viral replication in excised tissue; mph, month post hatching; Vp, phenotypic variance of the resistance trait; Vg, genetic or genomic variance of the resistance trait; FS, full-sib families; DH, doubled homozygous fish; BC, back-cross; SNP, single-nucleotide polymorphism; QTL, quantitative trait loci; cM, centi-Morgan; im, intramuscular; ic, intracelomical. Size of detected QTL correspond to either confidence intervals when calculated or cumulated windows with significant SNPs.*

In those studies, fish were either experimentally infected by immersion (29 studies) or by intraperitoneal, intramuscular, or intracelomical injections (27 studies). In six studies (Grimholt et al., 2003; Moen et al., 2009; Palaiokostas et al., 2018b; Robledo et al., 2018; Saura et al., 2019; Tadmor-Levi et al., 2019), infection was performed through cohabitation with previously infected fish. Finally, resistance phenotypes after a natural outbreak in farms were analyzed in two studies (Houston et al., 2008; Fraslin et al., 2019). Four studies combined resistance traits measured after two types of infection: natural outbreak and injection (Boison et al., 2019a), cohabitation and injection (Gonen et al., 2015), or immersion and injection (Fraslin et al., 2018; Rodríguez et al., 2019). While cohabitation or immersion routes are expected to be more representative of the natural route of infection and challenge the barriers to pathogen entry in the host, injection warrants the administration of a given dose of pathogen and allows addressing other (later) resistance mechanisms.

The main resistance trait investigated was the binary survival (BS, dead *vs.* alive status of the fish at the end of the challenge, in 24 studies), which is the principal outcome of infection and a relatively easy trait to measure. In many cases, this phenotype was refined with the analysis of time to death (TTD), measured as the interval between infection and death (analyzed alone in 5 studies and with BS in 23 studies). In three studies, a survival analysis using a Cox model that combined information of TTD and BS was performed (Verrier et al., 2013a; Fraslin et al., 2018; Saura et al., 2019). When possible, for all diseases caused by parasite and some viral diseases, the record of pathogen load was used as a resistance trait (in nine studies). Disease resistance was also assessed using a lesion score on gills for the amoebic gill disease (AGD) (Robledo et al., 2018; Boison et al., 2019b) or on heart for the cardiomyopathy syndrome (CMS) (Boison et al., 2019a; Hillestad and Moghadam, 2019). Some studies were able to refine resistance traits based on the appearance of disease signs and on detailed information about fish fitness. Endurance was measured as the time interval between the infection (Fraslin et al., 2018) or the first disease sign (Saura et al., 2019) and the death of the fish. In Saura et al. (2019), they also distinguished between resistance (time interval between infection and first disease sign) and resilience (measured as time between infection and death). Reliable and quantifiable resistance phenotypes are key for QTL analysis. In practice, detailed measurements of resistance (as survival, pathogen load, lesion score, or TTD) require a lot of effort (monitoring of fish several times a day) and are costly to perform on a high number of fish, thus limiting the detection power of QTL mapping studies. For an extensive comment on how to measure different resistance traits and the different types of experimental challenges or natural outbreak used in aquatic species, one could refer to Robinson et al. (2017).

The fish used in the majority of the studies presented in this review are half- or full-sibs from commercial populations produced by factorial mating designs (48 studies). In some studies, fish were produced using parents from lines with divergent phenotypes, by simple cross (F1 or F2 in 13 studies), backcross (BC in 9 studies) or gynogenesis, producing doubled haploid fish (DH in 2 studies). Those fish with divergent phenotypes can come from either lines that have been divergently selected for generations (e.g., Ozaki et al., 2001; Vallejo et al., 2014a, b) or from isogenic lines. When available, isogenic lines are useful tools to investigate molecular bases of complex traits as the genetic homogeneity within a line allows the repetition of measurement on the same genetic background through time and in different environments. When multiple isogenic lines are available, the genomic variability between lines is important [theoretically twice the variability of the population they were produced from Falconer and Mackay (1996)] and they can exhibit a wide range of contrasted phenotypes useful for QTL detection studies. However, in practice, only a few lines are available, reducing the variability range available for such studies. The use of doubled haploid fish (fully homozygous) increases the power of genetic analysis for a given number of fish (Martinez et al., 2002) but limits the fine mapping of QTL using LA approaches, detecting wide QTL. However, the production of DH fish is complicated to achieve with low survival rates (Komen and Thorgaard, 2007) and thus was only used in a limited number of studies on rainbow trout (Verrier et al., 2013a, 2018; Fraslin et al., 2018). Selection of fish from families with extreme phenotypes is a well-known approach to increase QTL detection power (Lander and Botstein, 1989) that has been used in many studies (e.g., in Houston et al., 2008, 2010; Geng et al., 2015; Palti et al., 2015; Wang et al., 2019).

Until 2014, the majority of QTL mapping studies were performed using LA approaches with a few hundred microsatellites markers and detected QTL locating within large confidence intervals on chromosomes. Such QTL contained hundreds of positional genes being potential candidate genes (Moen et al., 2007; Houston et al., 2008; Massault et al., 2011; Rodríguez-Ramilo et al., 2011; Vallejo et al., 2014a). From 2015, with the development of genotyping by sequencing techniques (Robledo et al., 2017) and of medium- to high-density SNP arrays in aquaculture species, tens or even hundreds of thousands of SNPs could be produced. Their use allowed an important narrowing of the confidence intervals of QTL detected by GWAS to less than hundreds of bp (Wang et al., 2017; Shi et al., 2018; Kong et al., 2019). This constitutes an important step regarding the use of QTL in selection and, together with the continuous improvement of the quality of genome sequence assembly in a number of species, in the identification of genetic variants involved in individual resistance variation.

### Resistance Mechanisms Hypothesized From QTL Analyses in Fish

The identification of underlying mechanisms from QTL analysis is notoriously difficult as QTL mapping identifies polymorphisms associated with a trait that can be distant from the causal mutation. Immune mechanisms are obvious targets when looking for resistance mechanisms, but the complexity of immune response and the high number of immune genes greatly complicate the task. Additionally, pathogens (especially intracellular ones such as mycobacteria or viruses) interact with many cell proteins that are not necessarily connected to immunity, but may affect the susceptibility of the host. In the following subsection, we will comment on a few QTL studies that pointed toward adaptive (see also Table 1) or innate mechanisms (see also Table 2). However, in most studies, even when QTL have been identified, the precise underlying mechanisms remain unknown (see also Supplementary Table 1).

#### QTL Pointing Toward Adaptive Mechanisms of Resistance/Susceptibility

Specific recognition of antigens expressed by pathogens by lymphocyte receptors diversified through VDJ somatic rearrangements of their genes is the archetype of adaptive response. During lymphocyte differentiation, immunoglobulin or T-cell receptor (TCR) loci are subjected to random genomic rearrangements of V, D, and J genes, resulting in the expression of a unique antigen (Ag) receptor by each lymphocyte. During the differentiation of lymphocytes, selective antigen-specific processes lead to elimination of most autoreactive cells and to proliferation of mature T and B cells. Clones specific to pathogen epitopes are expanded during antigen-driven responses. While immunoglobulins can recognize free native Ag, TCRs are specific for peptides that are processed by the antigen processing pathways in specialized cells and that are presented at the surface by MHC (major histocompatibility complex) molecules. The diversity (i.e., polymorphism) of the peptide binding groove of MHC molecules determines the repertoire of peptides that can be made available to T cell responses. Hence, variations in resistance to pathogens depending on adaptive responses may correspond to variations (1) of Ag receptor genes, (2) of the machinery mediating the recombination, (3) of genes affecting lymphocyte biology, or (4) of genes affecting Ag processing or presentation, including *mhc* genes. The large variation of *mhc* genes and the specificity of the effects of such variations make them targets of choice for pathogen-specific resistance mechanisms. Table 1 summarizes the relevant QTL studies pointing toward the implication of adaptive mechanisms in disease resistance (also, reviewed in Yamaguchi and Dijkstra, 2019).

##### Resistance associated to MHC

By genotyping polymorphic repeats located within the 3′UTRS of MHC class I and MHC class II genes, Grimholt et al. (2003) found highly significant associations between MHC class I (UBA) and class II (DAA-DAB) gene polymorphism and resistance to both a virus [infectious salmon anemia virus (ISAV)] and a bacterium (*Aeromonas salmonicida*) in Atlantic salmon. This was one of the first studies to establish the functional role of MHC molecules in disease resistance in fish, pointing toward adaptive mechanisms. A few low-resolution QTL mapping studies followed in Salmonids, specifically targeting MHC. Detailed information on the experimental design is available in Table 1 and Supplementary Table 1. In rainbow trout, Ozaki et al. (2007) performed a linkage analysis (LA) using 226 marker loci that included MCH class Ia, Ib, and II genes, genotyped in 199 individuals. They detected nine QTL associated with IPNV (infectious pancreatic necrosis virus) resistance and showed that only MHC class Ib gene mapped into a QTL, but finer mapping was not performed. Investigating Atlantic salmon resistance to sea lice, Gharbi et al. (2009) restricted their QTL analysis to the two linkage groups containing MHC class I and MHC class II genes but failed to show a proximal effect of MHC regions on salmon lice abundance. In turbot, Rodríguez-Ramilo et al. (2013) detected several QTL associated with resistance and survival time to the parasite *Philasterides dicentrarchi* (causative agent of scuticociliatosis). This study was based on 211 microsatellites and included the mapping of the MHC II B gene in the reference turbot map. Interestingly, MHC II B mapped within the confidence interval of one of the QTL, supporting this gene as being a candidate gene requiring further investigation. However, due to the low number of markers (average of 3.6–8.1 markers per linkage group), the detected QTL were associated with wide confidence intervals. In red sea bream, Sawayama et al. (2017) constructed low-density sex-specific genetic maps and mapped a candidate gene (MHC class II B). The QTL analysis for resistance to RSIV (red sea bream iridovirus) detected one major QTL, closely located to MHC class II B candidate gene. Although, in those studies, further mapping/association studies would be often required to definitively confirm that it is indeed the *mhc* genes that are involved.

##### Adaptive immunity is likely involved in the resistance to piscine myocarditis virus in Atlantic salmon

CMS, caused by piscine myocarditis virus (PMCV), is a severe inflammatory cardiac disease that affects farmed Atlantic salmon during the seawater production stage. With no commercial vaccine efficient against the virus, this disease causes major losses to the Norway salmon aquaculture industry (Hillestad and Moghadam, 2019). Two independent studies pointed toward the same genomic regions associated with resistance to the disease in different salmon populations from Norway, with several candidate genes shared and identified as involved in adaptive immunity (Boison et al., 2019a; Hillestad and Moghadam, 2019; see Table 1 and Supplementary Table 1 for details). Boison et al. (2019a) analyzed three populations from the Mowi breeding program. Resistance was assessed as BS for two populations infected during a natural outbreak, whereas in the absence of mortality in a third population (experimentally challenged by intraperitoneal injection of PMCV), resistance was assessed using a histology score of heart tissue. Hillestad and Moghadam (2019) analyzed a single population from SalmoBreed, challenged by intraperitoneal injection. As no mortality occurred during the challenge, viral loads were estimated in heart tissue by qRT-PCR and used as the resistance phenotypes. In the two studies, more than 500 fish (and up to 4300) per population were genotyped with a 55K SNP array. Despite being based on different genetic backgrounds and investigating resistance measured as different traits (BS, histopathological score, or viral load), both studies detected QTL located on the same two chromosomes, Ssa12 and Ssa27, suggesting that resistance to PMCV is controlled by similar regions within different populations. Interestingly, in both studies, GWAS performed on a finer phenotype detected QTL on SSa27 that were more significant and explained a higher proportion of genetic variance: 31.7% (based on histology scores of heart tissue) compared to 7.9–14% (based on BS) in Boison et al. (2019a); 57% (based on viral load) in Hillestad and Moghadam (2019). A fine phenotype might better reflect the true impact of the virus than BS and thus better reflect the true resistance of fish. Those studies highlight the importance of fine phenotyping for QTL mapping. Further examination to identify potential candidate genes involved in resistance to PMCV focused on 50 kb regions in Boison et al. (2019a) and on a 2 Mb-block in Hillestad and Moghadam (2019). On Ssa12, positional candidate genes involved in adaptive immunity included the *magi1* gene, known to impact the pathogenicity of coxsackievirus and adenovirus by regulating the amount of *car* receptors, in Boison et al. (2019a); two putative *MHC II antigen* genes and the *T cell transcription factor EB-like* gene (*tfeb*) in Hillestad and Moghadam (2019). On Ssa27, the regions investigated contained several genes with functions in antigen processing and presentation (both studies), as well as genes involved in the inhibition of viral cell replication (Boison et al., 2019a). Finally, Hillestad and Moghadam (2019) corroborated their findings with a previously published transcriptomic study (Timmerhaus et al., 2011) and showed that some of the genes identified by GWAS were differentially expressed between PMCV-infected and non-infected animals, reinforcing the potential role of adaptive immune response in Atlantic salmon resistance to PMCV.

#### QTL Pointing Toward Innate or Intrinsic Candidate Mechanisms of Resistance or Susceptibility to Infections

Beside adaptive responses, pathogens are involved in a large number of interactions with their host. The so-called “innate” immune response comprises a wide collection of defense mechanisms based on factors encoded in the genome of the host (i.e., which are not subjected to somatic diversification like B or T cell receptors). Genes directly involved in innate immunity typically play a role in pathogen sensing (such as Toll-like receptors and RIGI-like receptors), in signaling pathways responsible for transduction from sensors to effectors mechanisms (such as Myd88, NFkB), or in the effector phase of the responses that kills or inhibits pathogens (such as lysozymes, cathepsin, chitinase, complement, agglutinins, precipitins, etc.). Such genes can significantly affect host susceptibility to a pathogen. However, host resistance or susceptibility is determined by a much wider repertoire of genes: any host protein involved in an important interaction during the pathogen cycle may influence the efficiency of the infection. Hence, innate or intrinsic (i.e., expressed at steady state) mechanisms potentially underlying QTL of resistance to infections are extremely diverse, which makes understanding of underlying mechanisms very difficult. Of course, specialized defense pathways such as the type I IFN system for viral infections constitute *a priori* relevant targets for the corresponding pathogens. Table 2 summarizes the relevant QTL studies pointing toward the implication of innate or intrinsic mechanisms in disease resistance.

##### Many QTL studies typically detect multiple, quite wide QTL containing a number of genes potentially relevant for innate immunity

We present hereafter three examples: resistance to *Neoparamoeba perurans* (causative agent of AGD) in Atlantic salmon (Boison et al., 2019b), to *Flavobacterium psychrophilum* (causative agent of bacterial cold water disease, BCWD) in rainbow trout (Fraslin et al., 2018), and to viral hemorrhagic septicemia virus (VHSV) in turbot (Rodríguez-Ramilo et al., 2014). Details of experimental design and results are presented in Table 2 and Supplementary Table 1. QTL detected in those three studies were quite wide, with one QTL spanning up to 3 Mb in Boison et al. (2019b) and QTL with confidence intervals of up to 30 cM in Rodríguez-Ramilo et al. (2014) or even 100 cM in Fraslin et al. (2018), due to the relatively low number of fish and markers. Therefore, the number of genes within those QTL was important, and in order to detect candidate genes among them, the authors combined QTL detection with transcriptomic or comparative genetics approaches. Eventually, those studies evidenced the role of 20, 14, and 6 genes potentially relevant for innate response to AGD, BCWD, and VHS, respectively. Boison et al. (2019b) performed a GWAS to investigate Atlantic salmon resistance to *N. perurans*, using 53K SNPs genotyped in 1333 smolts challenged by immersion. Resistance was assessed using a qualitative scoring of gill lesions, and a transcriptomic analysis of naïve and infected fish was used to narrow the candidate genes’ list. In total, they detected three significant QTL as well as other suggestive QTL. Within the wide confidence intervals of the three significant QTL, they localized more than 20 genes including genes from the cadherin family, as the *protocadherin Fat 4* gene, and genes involved in the proinflammatory response of cytokine, as the *interleukine-18-binding protein* gene. In Fraslin et al. (2018), we detected QTL associated with rainbow trout resistance to *F. psychrophilum* after both immersion and injection experimental challenges using 310 doubled haploid fish from isogenic lines with contrasted resistance to the disease and genotyped for 2K RADseq SNPs. In this study we detected 15 QTL associated with resistance traits with wide confidence intervals (from 20 up to 100 cM). Interestingly, only three QTL were common between the two infectious routes, which suggests a more or less important role of certain mechanisms according to the mode of infection, or the existence of resistance mechanisms that are only triggered by one infection route. Using a previous study on the transcriptome response to infection in two isogenic lines of trout (Langevin et al., 2012), we identified 14 immune-related genes located within or close to the confidence interval of the QTL. The list included several genes involved in proinflammatory cytokine pathways, several interferon-stimulated genes, a TLR, an antimicrobial peptide, and the *c3* gene from the complement cascade. Interestingly, even if the study was performed on 5-month-old fish that already have a mature immune system with B and T cells, we did not detect genes belonging to the adaptive immunity. In Rodríguez-Ramilo et al. (2014), turbot resistance to VHSV was investigated by a low-density LA approach performed in 270 fish. Eleven suggestive QTL associated with TTD and/or BS were detected. Interestingly, one QTL was located within the LG20 that was syntenic with Omy3, previously detected as a major QTL associated with VHSV resistance in trout (Verrier et al., 2013a) and also pointed to the *tlr*7 gene. Further, to identify other candidate genes associated with resistance, they used a 0.5- to 1-Mb window around markers within syntenic blocks between *Gasterosteus aculeatus* and turbot. They detected some genes related to innate immune response such as *sugt1* coding for a protein with an essential role in the activation of Nod-like receptor that recognize invasive bacteria and *zc3hav1* coding for a protein related to induce innate immunity to viral infection. They also detected genes involved in apoptosis and in macrophage differentiation.

##### A few studies detected a major resistance QTL, explaining a large proportion of the genetic variance associated with positional candidate genes pointing toward the involvement of innate or intrinsic mechanisms

We present hereafter two studies as examples of major QTL detected with no causative mutation evidenced: resistance to VHSV in rainbow trout (Verrier et al., 2013a) and to *P. dicentrarchi* in turbot (Saura et al., 2019). We also develop the case of resistance to infectious pancreatic necrosis virus (IPNV) in Atlantic salmon (Houston et al., 2008, 2010, 2012; Moen et al., 2009; Gheyas et al., 2010), the only example of disease resistance study in fish in which a candidate causative mutation was evidenced (Moen et al., 2015). In Verrier et al. (2013a), a low-density linkage analysis approach performed on two families of doubled haploids trout allowed the detection of seven QTL associated with resistance to VHSV, including a major QTL on chromosome Omy3 also associated with viral replication in excised fin tissue infected *in vitro* (VREFT). This major QTL on Omy3 explained 33–49% of VREFT and 44–65% of TTD phenotypic variances. Several genes involved in innate immunity were located in the region of this major QTL, including Toll-like receptors 7 and 8 (Palti et al., 2010), cytokine receptors, and a trim (Verrier et al., 2013a). Functional studies performed in parallel demonstrated the involvement of the type I IFN pathway, as described below (see section *“*Isogenic doubled haploid lines, a tool of choice to unravel the complexity of host response to different pathogens and their interactions*”*). Saura et al. (2019), performed a QTL detection on 1800 turbot infected by cohabitation with *P. dicentrarchi*, a causative agent of scuticociliatosis. Using 18K SNPs, they detected one QTL associated with endurance (defined as the time between the first disease sign and the death of the fish) and four QTL associated with TTD (named resilience in the study) including a major QTL explaining 33% of the genetic variance of the trait. They performed a functional enrichment of candidate genes in this major QTL using the turbot transcriptome as a reference and detected 32 immune-related genes involved in tissue regeneration, response to wounding, inflammatory response, activation of NF-kappa-B pathway, and several genes involved in the activation of the innate immune response (activation of the TLR21 or the MAPK signaling pathways for example).

The case of IPN (infectious pancreatic necrosis) caused by IPNV in Atlantic salmon (*Salmo salar*) is one of the most famous example of identification of a major QTL associated with disease resistance in a fish species, potentially explained by the variations of an epithelial cadherin that prevents the binding and entry of the virus in the host cell. A major QTL was detected on the chromosome Ssa26 (linkage group LG21) in pre-smolt (2 mph) and post-smolt (10–13 mph) Scottish (Houston et al., 2008, 2010, 2012; Gheyas et al., 2010) and Norwegian (Moen et al., 2009, 2015) salmon populations. The markers associated with the resistant genotype are now routinely used in marker assisted selection by salmon breeding companies in Norway (AquaGen) and Scotland (Hendrix genetics, former Landcatch Natural Selection Ltd) for which IPN is no longer a concern. In Houston et al. (2008), this major QTL was first detected on post-smolts and explained up to 79% of the phenotypic variance in four segregating families. As the marker density was low (two to eight markers per linkage group), the confidence interval of this QTL was quite wide (10 cM). Later studies conducted on different Scottish sub-populations and including more fish and more markers (Houston et al., 2010, 2012; Gheyas et al., 2010) detected the same major QTL at a younger stage (2 mph) and were able to narrow the confidence interval down to 2 cM. The same QTL was detected at two stages (pre- and post-smolt) in a Norwegian population (Moen et al., 2009). This major QTL explained 83% of the genetic variance and 29% of the phenotypic variance of BS and had a confidence interval of 4 cM. However, in all these studies, they identified neither the causal mutation nor the underlying genetic resistance mechanisms but hypothesized the role of innate immunity as this major QTL was detected at young stage (2 mph), potentially before the development of an adaptive response (Zapata et al., 2006). By sequencing 22 homozygous resistant fish and 23 homozygous susceptible fish, Moen et al. (2015) identified a putative functional mutation within the cadherin domain of the epithelial cadherin gene (*cdh1-1*), causing a serine-to-proline amino acid shift. They confirmed the interest of this *cdh1-1* gene by targeting it in a functional approach using an immunofluorescence analysis in liver tissue showing IPNV binding to Cdh1-1 to enter the cell and infect the host. Thus, they hypothesized that resistance to IPNV in Atlantic salmon seemed to be explained by the virus failing to enter host cells due to *cdh1-1* mutation. However, while this putative functional mutation was in high linkage disequilibrium with three other SNPs strongly associated with resistance, it did not entirely correspond to the genotypes at the QTL. They hypothesized the existence of a second causative polymorphism involved in a two-locus model controlling resistance to IPNV. The fact that the *cdh1-1* mutation is the causative variant associated with salmon resistance to IPNV is still being debated as two later studies (Reyes-López et al., 2015; Robledo et al., 2016) reported that IPNV could replicate within resistant fish and thus could infect host cells, underlying the existence of a different causative mutation driving resistance to IPNV, still undiscovered.

#### Lack of Identification of Candidate Genes for Underlying Mechanisms of Resistance Can Be Due to Multiple Factors

In many cases, QTL mapping studies established a long list of positional candidate genes (i.e., genes located within QTL regions) but were not able to narrow down the list of immune-related genes to point toward innate or adaptive immunity, either because QTL were too wide or because they detected too many QTL. In other studies, no candidate genes were identified because no or poor quality genome assembly and genome annotation were available at the time of the publication. Supplementary Table 1 presents detailed experimental design and results of studies that identified QTL associated with resistance in various fish species but in which no immune mechanisms could be investigated or hypothesized.

In many studies, no functional candidate genes or underlying immune mechanisms could be proposed to explain the fish resistance because the detected QTL were too wide (sometimes corresponding to the whole linkage group). Firstly, the important size of the detected QTL might be due to a small number of markers used to produce a genetic map not dense enough to refine the QTL position, that could correspond to the whole linkage group as in Moen et al. (2004), Massault et al. (2011), Wiens et al. (2013), and Liu et al. (2016a). An example of refinement of QTL position by increasing the number of markers is the case of Asian sea bass resistance to nervous necrosis virus (NNV). Liu et al. (2016a) used 149 microsatellite markers to detect wide QTL (up to 13 cM) associated with both BS and TTD. In a following study performed on the same fish population genotyped with 3K SNPs, Liu et al. (2016b) were able to narrow some confidence interval to 1 cM and thus identified 62 positional candidate genes. With the development of genotyping by sequencing techniques as well as medium- to high-density SNP arrays, the number of markers used will no longer be the limiting factor. Secondly, increasing the number of genotyped individuals remains critical to refine the distances between markers on the genetic maps (dependent on the recombination rate) and to increase the analysis power for QTL detection. The production of an important number of fish is easier than in terrestrial species (as each fish can produce thousands of offspring); however, the recording of fine resistance phenotypes as well as the genotyping of an important number of fish is expensive and thus is becoming the main limiting factor for a powerful and fine mapping of QTL. Four studies performed GWAS with medium- to high-density SNPs arrays (31–690K) but using a relatively small number of fish (between 192 and 720), resulting in the detection of QTL with large confidence intervals: from 3 to 8 Mb for BCWD resistance in rainbow trout (Fraslin et al., 2019); from 27 kb to 3.7 Mb for resistance to enteric septicemia in catfish (ESC) (Zhou et al., 2017; Shi et al., 2018; Tan et al., 2018). No candidate genes’ list was established in Fraslin et al. (2019) because too many immune-related genes were positioned within those wide QTL. In the catfish studies, the list of potential genes involved in ESC resistance still contained many (more than 50) immune-related genes; thus, no clear mechanism of disease resistance could be inferred. In all those four studies, mapping of QTL along with gene expression study would have been needed to distinguish functional candidate genes among positional immune genes.

In other studies, no gene was identified because of the lack or poor quality of genome assembly or genome annotation available at the time QTL studies were published (Ozaki et al., 2013; Campbell et al., 2014; Vallejo et al., 2014b; Liu et al., 2015). High-quality genomic data and annotations are critical for identification of candidate causative mutations among significant SNPs detected by QTL approaches. Unfortunately, genome assembly is particularly difficult in many farmed fish species including salmonids and a number of cyprinids, due to recent whole genome duplications. Additionally, both the number of species of interest and the limited size of the fish biologists’ communities has hampered the progress of detailed functional annotation. Overall, the quality of the genomic information available is currently improving at a fast pace, thanks to the development of new sequencing technologies producing very long reads, and of new analysis tools. Also, the development of functional annotation initiatives, such as FAASG (Functional Annotation of All Salmonid Genomes), will be extremely important to speed up the progress of QTL approaches (Macqueen et al., 2017).

Finally, in other studies, because of the polygenic architecture of disease resistance traits, the authors did not investigate the positional genes located within the numerous medium or minor effect QTL detected. For example, Palaiokostas et al. (2018a) showed that sea bass (*Dicentrarchus labrax*) resistance to NNV is a trait controlled by at least seven QTL with minor to moderate effects on the trait (from 1.5 to 4% of genetic variance explained by each QTL). In Atlantic salmon, different studies also evidenced a polygenic architecture of the traits with an important number of QTL detected for resistance to *Gyrodactylus salaris* (Gilbey et al., 2006), *N. perurans* (AGD, Robledo et al., 2018), or sea lice (Tsai et al., 2016). Despite being a drawback when investigating the underlying mechanisms of a trait, polygenic architecture of disease resistance will not prevent the improvement of fish resistance using selective breeding. If the traditional pedigree-based selective breeding can be used to improve disease resistance in the absence of a major QTL (Sonesson et al., 2011; Gjedrem and Rye, 2018), genomic selection will usually perform better as it better takes into account the within-family variation (Ødegård et al., 2011). In those studies on VNN, AGD, and sea lice, the authors estimated that genomic prediction would result in a 10–27% increase in the accuracy of estimated breeding values of fish, more efficiently sorting fish between resistant and susceptible than with only pedigree information.

In summary, many studies in diverse fish species have investigated the genetic determinism of resistance to bacterial, viral, or parasitic diseases using QTL mapping or GWAS analyses. The large majority evidenced a clear genetic control of resistance traits, but to date, even when a major QTL controlling resistance was detected, only one study (Moen et al., 2015) could pinpoint a putative causal mutation that is still being debated. Yet, in a few cases, substantiated information about underlying mechanisms (i.e., role of receptor for IPNV in salmon, role of innate immunity for VHSV in trout) were obtained. In the next section, we will focus on transcriptomics and functional approaches aiming at deciphering molecular mechanisms involved in host–pathogen interactions.

## Host–Pathogen Interaction Mechanisms: Selection of Functional Candidate Genes Based on Transcriptomics and Functional Assays

### Transcriptomics and the Main Innate Responses to Pathogens: Building a Framework of Fish Responsiveness to Pathogens

Gene expression profiling is one of the possible approaches to get insight into the mechanisms explaining differential resistance to pathogens. Since high-throughput technologies have made possible comprehensive transcriptome description, one would expect that such analyses would, by comparing resistant (R) vs. susceptible (S) individuals, point toward defense mechanisms or pathways required for immunity. When performed on cohorts of significant size, such approaches can identify genetic variations potentially associated with severe or mild forms of the disease (Yamagishi et al., 2014). Furthermore, it is also crucial to understand how the host and the pathogen influence one another, particularly for complex pathogens such as bacteria or protozoans; transcriptome analyses can prove critical to shed light on such interactions.

In fish, transcriptome (and proteome) responses induced by viral or bacterial infections have been extensively studied in multiple tissues and species (reviewed in Verrier et al., 2011; Salinas, 2015; Benard et al., 2016; Martin et al., 2016; Ye et al., 2018), providing a rich resource for comparison. Still, transcriptome studies comparing resistant and susceptible genetic backgrounds have been performed only in a limited number of models. It is also important to note that one should keep in mind that the key factor for resistance may not be directly associated with large differential gene expression (i.e., below the detection threshold). The transcriptional response of rainbow trout to *F. psychrophilum* was compared between isogenic lines of rainbow trout with contrasted susceptibility to the infection (Langevin et al., 2012). The pronephros transcriptome was analyzed using micro-arrays 5 days after infection and showed a typical immune response involving antimicrobial peptides, complement, cytokines, and matrix enzymes. Key genes of the inflammatory response were more induced in susceptible animals where the bacterial load was also much higher. Although a complement C3 gene showed stronger induction in the resistant fish, it did not show an obvious contribution of the complement cascade to the variation of susceptibility to the infection. Analyses also revealed an extensive divergence between the transcriptomes of non-infected resistant and susceptible fish. A RNAseq whole-body transcriptome study was performed on resistant and susceptible fish produced by three generations of selection, comparing naïve and infected fish 1 or 5 days post-infection (Marancik et al., 2015). At both time points, the transcriptional response of resistant and susceptible fish was very different, with a large majority of genes up- or down-regulated only in one line. The expression of long non-coding RNAs (lncRNAs) was also analyzed in the same context (Paneru et al., 2016). Pairwise analyses between relevant conditions identified 556 differentially expressed lncRNAs. The pattern of correlation between differentially regulated lncRNAs and protein-coding genes suggested that some lncRNAs might control the expression of protein-coding genes involved in immunity and susceptibility to the pathogen. Differences between immune cell subsets from resistant and susceptible lines of rainbow trout have also been observed (Zwollo et al., 2017; Moore et al., 2018). For example, neutrophil-like cells expressing high-level Q4E (a trout neutrophil marker), MPO, Pu1, EBF, and IL1 were twice as abundant in the spleen of susceptible line compared to the resistant line. Also, fish from the resistant line have more IgT^+^ B cells in their spleen, pronephros, and blood than fish from the susceptible line. Further studies are required to clarify whether correlations between fish resistance to *F. psychrophilum* and the frequency of particular cell subsets mean that these cells play a role in the response to the infection.

In the case of *Piscirickettsia salmonis* in Atlantic salmon, deep sequencing of pronephros transcriptome revealed an exacerbated innate response in susceptible fish, while a few antibacterial genes like C-type lysozyme were more induced in resistant fish and may be involved in the resistance (Dettleff et al., 2015).

Two studies investigated the response of Atlantic salmon to the birnavirus IPNV, in the pronephros (Reyes-López et al., 2015) and in fry (Robledo et al., 2016). Surprisingly, in the first study, in susceptible families, many differentially expressed genes at day 1 returned to basal values at day 5 post-infection, while in resistant families, unlike susceptible families, most induced genes remained highly expressed at day 5. In the second study, while significant viral titer was observed in both resistant and susceptible fish at the analyzed time points, the inflammatory and cytokine response was stronger in susceptible fish, while resistant fish responded less with a noticeable induction of genes of the M2 macrophage pathway.

Transcriptome responses to the infection were also analyzed in resistant and susceptible fish to a few other viruses: VHSV in rainbow trout (Verrier et al., 2018, see below), herpesvirus CaHV in gibel carp (Gao et al., 2017; Mou et al., 2018), ISAV in Atlantic salmon (Dettleff et al., 2017), and Singapore grouper iridovirus in orange-spotted grouper (Yang et al., 2019).

Similar approaches were also followed with fish showing contrasted levels of resistance to parasites. For example, transcriptomic responses of the anterior kidney and skin of salmon with different levels of susceptibility to salmon louse (*Lepeophtheirus salmonis*) were compared (Sutherland et al., 2014; Braden et al., 2015; Holm et al., 2015). Studies comparing different salmon species (Sutherland et al., 2014; Braden et al., 2015) showed that species-specific pathways can be associated with resistance or susceptibility. Early proinflammatory TH1-type pathway appears to be generally important, but a later regulatory TH2-type response was observed in the skin of resistant coho salmon. Comparing low and high responding families of Atlantic salmon, Holm et al. (2015) observed higher expression of immune genes in resistant fish, suggesting that immunosuppression could explain susceptibility.

Overall, these studies have contributed to build a framework of fish responsiveness to a number of pathogens. It is very important to understand what pathways are differentially expressed in resistant and susceptible fish, to point to putative causal mechanisms and genes. Since several resistance mechanisms can act in combination, and pathogens can be highly variable, such global profiling is invaluable even when a main causal gene has been identified. However, such strategies also have significant pitfalls. It is often very difficult to distinguish variations that explain the extent and severity of infection from those that simply relate to its extent. Thus, the type I IFN response is a key factor to contain virus infection, but it is also a good indicator of the degree of the viral infection itself. The kinetics of transcriptome changes is often a critical parameter to identify resistance factors, but it is complicated to produce large temporal series of comprehensive datasets. Finally, the choice of tissues/organs analyzed is also difficult and can orientate toward genes relating consequences rather than causes of the resistance or susceptibility.

### Isogenic Doubled Haploid Lines, a Tool of Choice to Unravel the Complexity of Host Response to Different Pathogens and Their Interactions

Doubled haploid isogenic lines have been established in several fish species (Franěk et al., 2019). They offer unparalleled opportunities for a number of in-depth studies of complex traits. Indeed, for a given genetic background, it is possible to work with the whole organism, tissues, or derived cells at the same time, and with different pathogens or route of infection. These levels of investigation at different scales, and the comparison of different lineages, are very useful in dissecting the underlying mechanisms.

#### Isogenic Doubled Haploid Lines of Rainbow Trout: Susceptibility and Resistance to VHSV and the Type I IFN Response

A wide range of susceptibility to VHSV was observed among nine homozygous isogenic lines of rainbow trout produced by gynogenesis from a domestic population (INRA Synthetic strain, Quillet et al., 2007). While all these isogenic lines were susceptible to VHSV infection after injection, they exhibited a surprising diversity of susceptibility levels leading to final survival from 100 to 0% after waterborne infection, with some lines having intermediate positions. The virus was seldom found in the spleen, blood, or pronephros of resistant fish, confirming a resistant (not a tolerant) phenotype. To get insight into the mechanism of resistance to the virus, two functional assays were developed. First, *in vitro* cultures of fin explants (Quillet et al., 2001) from resistant and susceptible fish were infected by the virus to assess their capacity to support viral production. The virus production was well correlated to the susceptibility level of the fish from which fins had been sampled. This correlation was further supported by the detection, in two different trout families, of a single major QTL governing both survival of fish after waterborne infection and virus production in *in vitro* cultures of fin explants (Verrier et al., 2013a). In parallel, fibroblast cell lines were developed from several isogenic trout lines (Verrier et al., 2012). Their susceptibility to VHSV infection was remarkably consistent with the susceptibility of the parental fish, indicating that resistance mechanisms associate with innate or intrinsic factors. Interestingly, comparison of the type I IFN response of the cell lines derived from the two fully resistant fish lines revealed two distinct mechanisms: the high resistance of one cell line (named B57) was largely due to an early interferon IFN induction, which was not observed in susceptible cells, while the other cell line (A02) was more refractory to infection—as the corresponding fish—due to different mechanisms.

Deep sequencing of the transcriptome of one resistant (the same B57 line) and one susceptible cell line after stimulation with inactivated VHSV revealed a stronger and earlier response in the resistant background (Verrier et al., 2018). Many typical interferon-stimulated genes were induced, such as *irf1*, *rsad2*, and members of the *ifi44*, *gig*, *parp*, *ifit*, and *cd9* families, as well as many fintrim genes. Also, several major factors of the antiviral jak/stat pathway were much more expressed in non-stimulated B57 cells compared to the susceptible cells, possibly contributing to faster antiviral response.

Altogether, these data illustrate that functional assays provide a better understanding of the pathways involved in resistance. The multiplicity of phenotypes underscores the complexity of the mechanisms, likely based on a similarly complex genetic architecture. Even if the functional assays and transcriptome analyses did not identify the causal gene(s), they have built a framework of the mechanisms and pathways involved in the response of resistant and of susceptible fish to VHSV. This knowledge is fundamental to fully understand the structure of the resistance as well as the possible subversive mechanisms evolved by the pathogen.

#### Isogenic Doubled Haploid Lines of Rainbow Trout: Specificity of Susceptibility and Resistance to Multiple Pathogens

The work of VHSV resistance pointed toward innate mechanisms, probably connected to the strength and kinetics of type I IFN response. One may expect that such factors or pathways may be generic and would mediate a significant level of resistance against other viruses. The fish line B57, which was highly resistant to VHSV, showed high to very high resistance levels to all tested viruses (Table 3). Consistent results were observed *in vitro* using the corresponding cell line. Interestingly, while our functional assays indicated that the line A2 was resistant due to other mechanisms, these fish were also highly resistant to IHNV. However, testing 10 different isogenic lines, no overall correlation between resistance to those two rhabdoviruses was observed (Verrier et al., 2013b). This seems to be the general picture, as further experiments with a number of isogenic lines indicated that resistance levels to different viruses are not generally correlated. For example, A36 was highly susceptible to VHSV and IPNV, while being rather resistant to IHNV and ISAV; in contrast, B3 was susceptible to IHNV and IPNV and more resistant to VHSV and ISAV (Table 3).

**TABLE 3:** Resistance and susceptibility of a collection of rainbow trout and cell lines against multiple pathogens.

Pathogen, strain, and route of administration	Genetic background								
	A2		B57		A36			B45	
	Fish	Fibr	Fish	Fibr.	Fish		Fibr.	Fish	Fibr.
VHSV, bath	R*		R		S			S	
VHSV, *in vitro*		R		R			S		I
IHNV, bath	R		R		R			S	
IHNV, *in vitro*		R		R					
IPNV, bath			R		S				
ISAV, bath			R		R			S	
^(a)^*F. psychrophilum* FRDGSA 1882/11, bath	R		R		S			I	
^(b)^*F. psychrophilum* FRDGSA 1882/11, IM	I		S		S			S	
^(b)^*F. psychrophilum* OSU THCO2-90, IM	R		S		S			R	
^(c)^*A. salmonicida* 36-75R, IM	n.d.		S		R			R	

**Pathogen, strain, and route of administration**	**Genetic background**								
	**A22**		**A3**		**AP2**	**B3**	**A32**	**Syn**	**RTG**
	**Fish**	**Fibr.**	**Fish**	**Fibr.**	**Fish**	**Fish**	**Fish**	**Fish**	**Fibr.**

VHSV, bath	S (0%)		S		S	R	R	S	
VHSV		S		S					S
IHNV, bath	S (17%)		R		S	S	S		
IHNV		S		S					
IPNV, bath	I (55%)		S		S	S	I	I	
ISAV	S (0%)		I			R			
^(a)^*F. psychrophilum* FRDGSA 1882/11, bath	S		R		R	R	R	R	
^(b)^ *F. psychrophilum* FRDGSA 1882/11, IM	S		I		R	R	I	n.d.	
^(b)^*F. psychrophilum* OSU THCO2-90, IM	S		I		R	R	n.d.	R	
^(c)^*A. salmonicida* 36-75R, IM	R		I		n.d.	S	S	n.d.	

*S, susceptible; R, resistant. ^(a)^Resistance/susceptibility phenotype estimation using the percentage of survival 28 days after immersion challenge. S: susceptible (0–30%), R: resistant (>70%), I: intermediate (30–70%). ^(b)^Isogenic lines were classified into three groups (S, susceptible; R, resistant; I, intermediate) based on the time to death (TD) values or % of survival after intramuscular injection challenge. TD of group S < TD of group I < TD of group R. ^(c)^Resistance/susceptibility phenotype estimation using the percentage of survival 13 days following the IM challenge. S: susceptible (0–30%), R: resistant (>70%), I: intermediate (30–70%). n.d., not done; Fibr, fibroblasts; VHS, viral hemorrhagic septicemia; ISAV, infectious salmon anemia virus; IHNV, infectious hematopoietic necrosis virus; IPNV, infectious pancreatic necrosis virus. Data are compiled from Quillet et al. (2007) [VHSV], Biacchesi et al. (2007) [ISAV], and Verrier et al. (2013b) [IHNV], or unpublished (*Flavobacterium psychrophilum*, IPNV).

This collection of isogenic lines was also challenged with two bacterial pathogens, *F. psychrophilum* (BCWD) and *A. salmonicida* (furunculosis), and contrasted resistance and susceptibility were observed between isogenic lines (Table 3, unpublished results). This character was largely influenced by the bacterial species, as well as partly by the route of bacterial infection and by the genetic background of *F. psychrophilum*. Two isogenic lines showed high susceptibility (A36, A22) or strong resistance (AP2, B3) toward *F. psychrophilum* infection, whatever the route or strain used for the challenge. However, for several isogenic lines, this trait was influenced by the route of infection, which may be indicative of different mechanisms of resistance in the different lines. For instance, B57 was highly susceptible by injection but gained in resistance by bath, while A2 and A3 showed intermediate resistance by intramuscular injection compared to bath. The existence of favorable genetic factors controlling defense mechanisms triggered after waterborne infection in line B57 was further supported by the results of a QTL analysis (Fraslin et al., 2018). The resistance/susceptibility status was also compared between fish challenged with two *F. psychrophilum* strains from different genetic lineages as defined by Multi Locus Sequence Analysis, namely, FRDGSA 1882/11 (clonal complex ST90) isolated from rainbow trout and OSU THCO2-90 (clonal complex ST9) isolated from Coho salmon (Duchaud et al., 2018), leading to opposite ranking for one isogenic line (B45) only. Some interactions between the host genetics and the bacterial genotype may occur and should be considered when selecting for BCWD resistance. This study also pointed to a negative relationship of the resistance character between *F. psychrophilum* and *A. salmonicida*, highlighting the importance of screening for resistance to various pathogens in breeding schemes.

Table 3 also illustrates that there is no straightforward correlation (or reverse correlation) between the resistance to viral and bacterial infections. These data illustrate the strong interest of comparing the resistance levels to multiple pathogens across stable genetic backgrounds. Within a given species, it confirms the complexity and diversity of genetic architectures of resistance to different viruses or bacteria. The strong impact of the route of infection provides further insight into the type of mechanisms involved and might deserve further investigation. Combined with QTL data, genome sequencing of isogenic lines will help determine the best candidate genes/sequence polymorphisms for mechanisms responsible for resistance and susceptibility. These models may also constitute an interesting context to test the interactions between susceptibility factors to different pathogens.

### Importance of KO Approaches *in vivo* and *in vitro*

In order to validate the role of a locus on a particular phenotype of resistance or sensitivity to a pathogen, loss-of-function methodologies are very valuable (Vidalin et al., 2009). For many years, the development of knock-out approaches was limited to animal models relevant to the medical research community, mainly mice (Hall et al., 2009). Today, with the advent of CRISPR/Cas9-based genome editing combined with an increasing number of well assembled genomes, it can be extended to farmed fish species (Yáñez et al., 2015). Proofs of concepts of efficient gene KO by CRIPSR/Cas9 have been established *in vitro* for chinook salmon cells (Dehler et al., 2016) or *in vivo* in Atlantic salmon (Edvardsen et al., 2014). However, the latter is faced with the difficulty to produce non-mosaic animals (Mehravar et al., 2019).

Cell lines can be valuable to verify the relevance of one or several candidate genes. In the case of certain traits such as innate immunity to viral pathogens (Langevin et al., 2019), phenotypes obtained in a KO cell line can be extrapolated to the whole animal with some degree of accuracy. This is the case of the type I interferon (IFN1) system, an important arm of the immunity to viruses. Dehler et al. (2019) generated a chinook salmon cell line (named GS2) with the gene encoding for Signal transducer and activator of transcription (*stat2*), an important molecule involved in the signaling of IFN1, disrupted. The transcriptomic characterization of the GS2 cells demonstrated that they had lost their ability to respond to IFN1.

In addition, the production of additional KO cell lines in the same species suggested that the CRISPR/Cas9 approach allows the generation of homozygous null mutations at one or several paralogous loci. This is particularly well suited to the European commercial fish species, salmonids, and carp, with a duplicated genome.

Genome-wide KO *in vitro* screen is also possible in well-established mammalian–lentivirus systems (Shalem et al., 2014, 2015) and may be applicable in fish cells in the near future (Gratacap et al., 2020). Genome editing can also target non-coding sequences and contribute to validation of mutation in, for instance, regulatory sequences (Banerjee and Sherwood, 2017; Kang et al., 2019).

Thus, *in vitro* knock-out could help validate phenotypes in a certain number of biological functions that can be accurately replicated in cell lines (such as antiviral innate mechanisms). In a second stage, *in vitro*-validated single guide RNA can be used to generate KO animals within the same species. Nuclear transfer from edited somatic fish cells to oocytes is a strategy that merits some future attention (Ju et al., 2003) in order to circumvent the current problems of mosaicism.

### Perspectives

QTL analyses draw a first picture of the genetic architecture of disease resistance. With the continuous development of new technologies allowing the genotyping of tens to hundreds of thousands of SNPs in larger datasets, the precision of the QTL position has dramatically increased in recent studies (confidence intervals reduced to sometimes tens of kb), with promising opportunities for the identification of underlying acting factors.

With a few exceptions, disease resistance in fish appears to be mainly controlled by several genomic regions having a minor to medium impact on the trait, underlying a complex determinism. In many studies, resistance was controlled by tens of minor or medium effect QTL, revealing the polygenic architecture of the trait. In others, a few QTL—sometimes major ones—that had a bigger impact were detected alongside with smaller-sized QTL, revealing an oligogenic architecture of resistance. Only a few examples of simple determinism were reported with one or two major QTL associated with Atlantic salmon resistance to IPNV (Houston et al., 2008, 2010, 2012; Moen et al., 2009, 2015; Gheyas et al., 2010) and PMCV (Boison et al., 2019a; Hillestad and Moghadam, 2019), red sea bream resistance to RSIV (Sawayama et al., 2017), and rainbow trout resistance to whirling disease (Baerwald et al., 2011).

Besides being often polygenic, disease resistance is also a complex trait for which detected QTL might often be in interactions with each other. Epistasis has been shown to play an important role in complex traits but is often neglected as it is complex to measure and take into account (Flint and Mott, 2001; Carlborg and Haley, 2004). Two studies have investigated epistatic interactions between QTL associated with disease resistance in fish. Fraslin et al. (2018) detected 15 QTL associated with resistance to *F. psychrophilum* in doubled haploid rainbow trout. In this study, by fixing the alleles at the five main QTL detected previously as co-factors in a new model, they were able to reveal five new QTL, underlying the polygenic and complex nature of rainbow trout resistance to *F. psychrophilum*. In Tadmor-Levi et al. (2019), they tested pairwise interactions between their QTL by including QTL effect as a fixed effect in a new model and detected multiple epistatic QTL associated with resistance to cyprinid herpes virus-3 (CyHV-3) in five common carp families. Interestingly, once again, some QTL that were not significant in families in a simple model became significant when epistasis was accounted for. In total, three types of interactions were evidenced, “enhancing-like or synergistic” interaction when the allele at one QTL enhances the effect of the allele at the second QTL, “compensation-like” interaction when each QTL alternatively contributes to the resistance and “nullifying” interaction when both QTL have opposite effects that cancel each other’s main effect. In Fraslin et al. (2018), they also evidenced “counter-acting interactions,” which might correspond to negative feedback loops, when the favorable or deleterious effects of the genotype at one QTL was reversed according to the genotype at the interacting QTL. While complicated to evidence, epistatic interactions between disease resistance might be common and underline the complexity of resistance mechanisms pathways.

When the QTL detected were not too wide and when an annotated genome assembly was available, those QTL studies also identified putative candidate genes located within the QTL, underlying complex immune pathways that pointed toward adaptive or innate immunity (see Tables 1, 2, respectively). However, even when a narrow list of positional candidate genes was established, only one study (Moen et al., 2015) identified the putative causative mutation.

GWAS approaches to map QTL and detect positional candidate genes can be completed with different approaches to refine both QTL location and candidate genes’ list by integrating genetic diversity, comparative genomics, or functional studies. GWAS, genetic diversity (*F*_st_), and nucleotide diversity filtration approaches can be combined to select the most promising positional candidate genes, as in Zhou et al. (2019) for the resistance to *Vibrio harveyi* in the Chinese tongue sole (*Cynoglossus semilaevis*): in this way, nine significant SNPs located in six candidate regions (23 putative candidate genes) were identified from an initial list of 79 regions. In a different perspective, Yáñez et al. (2019) combined GWAS approaches and comparative genomics to detect QTL associated with resistance to *P. salmonis*, an intracellular bacterium responsible for salmon rickettsial syndrome (SRS), in three salmonids species (Atlantic salmon, coho salmon, and rainbow trout). Using between 9K and 42K SNPs, they described an oligogenic architecture of the trait in rainbow trout and coho salmon with the top 200 SNPs explaining between 70 and 90% of genetic variance of BS or TTD in the two species. However, in Atlantic salmon, resistance to *P. salmonis* was a polygenic trait with no SNPs explaining over 5% of the genetic variance and the top 200 SNPs explaining 30% of both BS and TTD. To narrow the long list of positional candidate genes, they classified SNPs and corresponding genes into four categories according to (1) function of protein domain or (2) orthology among species, (3) proximity to the top SNP explaining the highest percentage of genetic variance, and finally (4) presence in more than one genomic region explaining more than 1% of the genetic variance within species. The list of best positional candidates involved in salmonids resistance to *P. salmonis* could be reduced to 21 genes belonging to at least two of those four categories. This study shows that comparative genomics can be an interesting approach to detect highly conserved resistance mechanisms to one pathogen among various fish species (here three salmonids); however, by doing so, species-specific mechanisms are not investigated. In three previous publications performed by the same group in Chile on rainbow trout (Barria et al., 2019), Atlantic salmon (Correa et al., 2015), and coho salmon (Barría et al., 2018), species-specific QTL have been identified, pointing to involvement of both innate and adaptive immunity in Atlantic salmon resistance to *P. salmonis*, and of innate immunity in rainbow trout and coho salmon (see Supplementary Table 1).

Recent studies combined QTL mapping with whole-genome sequencing (WGS) of pooled or single DNA samples, and expression analysis (RNAseq or qPCR) to search for candidates genes underlying disease susceptibility/resistance. Palaiokostas et al. (2018b) combined QTL mapping and WGS to refine the position of QTL of carp resistance to koi herpesvirus, and detected a promising positional and functional candidate gene. After performing WGS on two pooled DNA libraries of 30 susceptible and 30 resistant fish, they annotated new SNPs in the main QTL region, and identified a potential causal mutation responsible for a premature stop codon in the E3 ubiquitin ligase *trim25*, a major Interferon Stimulated Gene involved in RIG I and IFN signaling. The mutation responsible for the premature stop codon was rare, but more common in the susceptible fish than in the resistant fish pools. *trim25* gene therefore appeared as a promising functional candidate to explain resistance to koi herpesvirus in carp. In another study on the genetic architecture of Atlantic salmon resistance to sea lice (*Caligus rogercressey*), Robledo et al. (2019) succeeded to identify a few top positional and functional candidate genes by combining GWAS and transcriptomics study in the fish skin. Those genes included *tob1*, a transcription factor that negatively regulates cell proliferation, and the serine/threnonine-protein kinase 17B (*stk17b*) showing also the highest fold change between healthy and diseased skin.

These examples illustrate that future projects to understand the genetic bases of fish resistance to diseases will combine a number of approaches taking advantage of novel genomic and sequencing technologies.

eQTL (expression quantitative trait loci) and reQTL (RNA-eQTL) will certainly constitute another important approach to identify relevant causal genetic variants in fish as it is the case in mammals (Gat-Viks et al., 2013). Importantly, fast functional tests such as *in vitro* models and the powerful loss-of-function screens based on CRISPR/Cas systems will likely complement the arsenal used by fish immuno-geneticists to foster the development of integrated pipelines for fast identification of key genes regulating resistance to infections. When available, isogenic fish lines with contrasted phenotypes will provide interesting models to explore the mechanistic diversity of resistance, as illustrated by studies on VHSV in rainbow trout (Verrier et al., 2013a, 2018).

A largely unresolved question is how genetic resistance and susceptibility to pathogens may have an effect on, or may be correlated to, the efficiency of vaccination against these pathogens or others. There are multiple possible links between vaccine development and natural resistance to pathogens. First, resistance based on adaptive immune responses, in particular those determined by MHC haplotypes expressed by an individual, often depend on the structure of selected immune repertoires of lymphocytes, which favor efficient and protective responses. In such cases, it is likely that genetic resistance to a pathogen, and good responses to vaccination against the same pathogen, would be correlated. Robust vaccines would therefore have to induce protective responses even in susceptible animals naturally expressing repertoires devoid of the most relevant specificities for this response. Second, when the resistance to a pathogen is mediated by components of innate immunity-for example by pathogen sensing—it would likely impact the strength of immune responses induced by vaccines or even, more generally, by many adjuvants. Finally, the resistant or susceptible status of experimental fish is an essential parameter to take into account when testing the protective effects of a vaccine. Robust tests of vaccine efficiency require control groups with high levels of mortality upon challenge, but it might be also interesting to test the response induced in naturally resistant individuals. While it remains difficult to integrate such aspects in the vaccine development for the time being, a better understanding of the mechanisms of genetic resistance to pathogens should contribute to a better design of vaccines and adjuvants in the future.

## Conclusion

In this review, we focused on resistance and susceptibility to infections in fish. Upcoming progress in the field will certainly lead to a better understanding of the importance of endurance and tolerance/resilience (Yáñez et al., 2010; Levraud et al., 2014; Fraslin et al., 2018; Fraslin, 2018; Saura et al., 2019). Finally, a dynamic vision of robustness should emerge, in the context of a given aquatic farming system and its pathobiome.

## Author Contributions

CF, EQ, DL, and PB planned the review topic, reviewed the manuscript, and wrote and edited the manuscript. TR, ND, J-FB, and BC wrote specific sections of the manuscript, synthesized the information for specific tables, and reviewed and edited the manuscript. All authors read and approved the final manuscript.

## Conflict of Interest

The authors declare that the research was conducted in the absence of any commercial or financial relationships that could be construed as a potential conflict of interest.

## Abbreviations

Ag: antigen
AGD: amoebic gill disease
BCWD: bacterial cold water disease
BS: binary survival
CaHV: Carp herpes virus
CMS: cardiomyopathy syndrome
CRISPR: Clustered Regularly Interspaced Short Palindromic Repeats
eQTL: expression QTL
ESC: enteric septicemia in catfish
GWAS: genome-wide association studies
IFN: interferon
IPNV: infectious pancreatic necrosis virus
ISAV: infectious salmon anemia virus
INHV: infectious hematopoietic necrosis virus
KO: knock-out
LA: linkage analysis
lncRNA: long non-coding RNA
MHC: major histocompatibility complex
NNV: nervous necrosis virus
PMCV: piscine myocarditis virus
QTL: quantitative trait loci
reQTL: RNA-eQTL
SNP: single-nucleotide polymorphism
TCR: T-cell receptor
TH: T helper
TLR: Toll like receptor
TTD: time to death
VHS: viral hemorrhagic septicemia
VHSV: viral hemorrhagic septicemia virus
VNN: viral nervous necrosis.
